# Factors contributing to racial disparities in influenza vaccinations

**DOI:** 10.1371/journal.pone.0213972

**Published:** 2019-04-03

**Authors:** Suma Vupputuri, Kevin B. Rubenstein, Alphonse J. Derus, Bernadette C. Loftus, Michael A. Horberg

**Affiliations:** 1 Mid-Atlantic Permanente Research Institute, Mid-Atlantic Permanente Medical Group, Kaiser Permanente Mid-Atlantic States, Rockville, Maryland, United States of America; 2 Mid-Atlantic Permanente Medical Group, Kaiser Permanente Mid-Atlantic States, Rockville, Maryland, United States of America; University of Nebraska Medical Center, UNITED STATES

## Abstract

**Background:**

Racial/ethnic disparities in rates of influenza vaccinations in the US remain an issue even among those with access, no out-of-pocket costs, and after adjusting for confounders. We used an approach called the Oaxaca-Blinder (OB) decomposition method to ascertain the contribution of covariates individually and in aggregate to the racial disparity in influenza vaccination.

**Methods:**

We included members > = 18 years of age as of 05/01/2014 with continuous enrollment through 04/30/2015. Influenza vaccination was defined by diagnosis, procedure, or medication codes, or documentation in the immunization table. Characteristics were reported by race. Logistic regression models estimated the odds of vaccination associated with: (1) race; and (2) covariates stratified by race. The Oaxaca-Blinder (OB) method calculated the contribution of covariates to the difference or disparity in vaccination between Blacks and Whites.

**Results:**

We found that among adults, 44% were vaccinated; 55% were Black; and 45% were White. Black members have 42% lower odds of vaccination than White members. The contribution of the differences in the average value of the study covariates between Black and White members (the OB covariate effect) accounted for 29% of the racial disparity. The contributions to the total White-Black disparity in vaccination included: age (16%), neighborhood median income (11%), and registration on the online patient portal (13%). The contribution of the differences in how the covariates impact vaccination (OB coefficient effect) accounted for 71% of the disparity in vaccination between Blacks and Whites.

**Conclusion:**

In conclusion, equalizing average covariate values in Blacks and Whites could reduce the racial disparity in influenza vaccination by 29%. For health system vaccine campaigns, improving registration on the patient portal may be a target component of an effective system-level strategy to reduce racial disparities in vaccination. Additional information on patient-centered factors could further improve the value of the OB approach.

## Introduction

Influenza spreads in a yearly outbreak, resulting in three to five million cases of severe illness and about 250,000 to 500,000 deaths globally [1], and an annual average of 41,400 deaths in the U.S. [2] Influenza occurs with an annual attack rate of 5%–10% in adults [1, 3] and can result in hospitalization and death mainly among high-risk groups such as older persons, young children, persons with certain health conditions, and pregnant women [4]. Safe and effective influenza vaccines are widely available and have been used for more than 60 years.[5] [6]

Influenza vaccination coverage in the U.S. was 47.1% in 2014 and was lower among Blacks (43.8%) than Whites (48.5%).[7] Racial/ethnic disparities in rates of influenza vaccinations in the US remain an issue even among those with no out-of-pocket costs for the vaccination and after adjusting for confounders such as sociodemographic characteristics, health status, insurance, access, and geographic region.[8–13] Until recently, racial disparities in health outcomes or preventive services such as vaccinations have been assessed in relatively simple terms: (1) examining the rate of influenza vaccination in Blacks compared to Whites; or (2) assessing, in an etiologic model, the statistical significance and magnitude of the race term. When we do find a gap between Whites and Blacks in influenza vaccination, the question follows: how much of that gap can be explained by observable characteristics? An approach called the Oaxaca-Blinder decomposition method was developed to address this question. [14, 15]

Kaiser Permanente Mid-Atlantic States (KPMAS) is a group-model health maintenance organization that provides comprehensive, pre-paid health care to approximately 750,000 members in Maryland, Virginia, and the District of Columbia. KPMAS is composed of the Kaiser Foundation Health Plan and the Permanente Medical Group, a multi-specialty group practice that provides health care for members of the health plan. KPMAS has made annual influenza vaccination a priority initiative to prevent illness with a vaccination program consisting of no-referral flu vaccination clinics in all medical centers from September through late December; no copays; and a widespread education campaign. Despite these efforts, racial disparities in vaccination at KPMAS persist. In the era of “Learning Health Systems” (i.e. systems where internal data and scientific evidence are systematically integrated within system culture in order to use that knowledge for improving practice), the Oaxaca-Blinder methodology will specifically identify factors that demonstrate a strong contribution to racial disparities in influenza vaccinations in order to target these factors operationally. We aim to provide evidence and guidance to health systems on where to put resources and efforts to potentially reduce the disparity in influenza vaccination.

The purpose of this study was to examine whether the difference in the proportion of patients vaccinated at Kaiser Permanente Mid-Atlantic States (KPMAS) between Whites and Blacks (ie. the racial disparity) can be explained by the distribution and the impact of a comprehensive list of pre-specified covariates. In order to do this, we will assess: the distribution of covariates by race; the odds of vaccination associated with the covariates stratified by race; and finally, the meaningful contribution of the covariates; and the effects of those covariates on the racial disparity in influenza vaccination.

## Methods

### Data source

The study was conducted at Kaiser Permanente Mid-Atlantic States (KPMAS), an integrated health care delivery system that maintains comprehensive electronic medical records (EMRs). We used data from May 1, 2014 to April 30, 2015.

### Study population

We included members who identified as White or Black (either African-American or African immigrant), were 18 years of age or older on May 1, 2014, and who had continuous enrollment (allowing for 60-day gaps) between May 1, 2014 –April 30, 2015. A total of 236,511 KPMAS members met the inclusion and exclusion criteria. There were missing data for certain covariates including smoking status (17.3% missing) and provider race (5.3% missing). Neither smoking status nor provider race demonstrated differential missingness by patient race. However, most of the other covariates were missing at rates less than 1%. Overall, we included 184,745 patients in the regression analyses.

### Study variables

Race was defined by self-reported identification to a category of race. Our outcome was influenza vaccination, a dichotomous variable (yes/no) indicating influenza vaccination during the study period. The proportion vaccinated was calculated as the number of patients who received the vaccine divided by the total population at risk during the study period. Patients who self-reported flu vaccination outside of KPMAS were also included as vaccinated.

Data obtained from the EMR included demographics (age at the beginning of the study period, gender, race, service area); comorbidities (Charlson Comorbidity Index [CCI] [16]); health system information (distance to their KPMAS Medical Center); and behavioral factors (current cigarette smoker and being registered on “KP.org”—an online portal available to all KPMAS members where, if registered, they can access their medical record, appointments, and health education, including detailed information on influenza vaccinations). Demographic data at the neighborhood level were obtained by using patients’ geocoded addresses which were matched to census data at the block group level (neighborhood median household income). We used neighborhood-level median household income as a proxy for individual-level income. Charlson Comorbidity Index (CCI) was calculated to assess burden of comorbidities.[16] Finally, provider data was used to determine the race of patients’ primary care providers to examine discordant patient/provider race as a covariate.

### Statistical analysis

Summary statistics of the characteristics of the study population, including influenza vaccination status, were reported by race. P-values for the association between categorical variables with race and continuous variables with race were calculated using Pearson's Chi-squared [17, 18] and Welch's T-Tests [19–21], respectively. Logistic regression models assessed the odds of influenza vaccination associated with White and Black race, adjusting for *a priori*-specified covariates. This model determined the evidence for a racial disparity in influenza vaccination.

We then used an extension of the Oaxaca-Blinder (OB) decomposition method [14, 15, 22] for logistic models to calculate the contribution of the covariates described above to the disparity in vaccination by race. The OB method allows the assessment of the difference or disparity between two groups to be partitioned into both differences in observable characteristics/covariates, and the difference in the effects of those characteristics across groups. We want to establish not only that a disparity exists, but also quantify the extent that certain pre-specified covariates contribute to the disparity. The OB method was developed to study the gender wage gap. It enabled the authors to estimate the proportion of the wage gap attributable to differences in education, work experience, or other relevant characteristics, as well as the proportion that cannot be accounted for by these variables. This remaining proportion may be attributed to gender discrimination or unobserved predictors of wages.

To conduct the OB analysis of the disparity in vaccination rates, we used coefficients from logistic regression models stratified by race as well as average covariate values within each race to decompose the racial disparity into a **covariate** effect (the effect of differences in the average value of all covariates between the Black and White groups on vaccination), and the **coefficient** effect (the effect of differing impact of the covariates on vaccination between the Black group and the White group). We then estimated the relative contribution of each of the covariates to: (1) the covariate effect; (2) the coefficient effect; and (3) the total racial disparity. In other words, we are determining how differences in observable characteristics between the White and Black groups contribute to the overall racial difference in vaccination rates, and we are also determining how differences in the effects of those characteristics on vaccination between White and Black groups contribute to the overall racial difference in vaccination rates. It is important to note that these contributors to the racial difference/disparity may contribute via exacerbating the racial disparity or, alternately, reducing the racial disparity. (A description of a hypothetical counterfactual population and an informative example is given in S1 Text).

Data extraction and quality control were performed using SAS software (version 9.4; SAS Institute, Cary, NC). Descriptive statistics and logistic regression models were computed using R software (R version 3.1.1, Core Team 2013, Vienna, Austria). The Oaxaca-Blinder decomposition for logistic models was computed using the oaxaca command with the logit option in Stata (Release 14, StataCorp LP 2015, College Station, TX) [23].

This study was approved by the Institutional Review Board (IRB) at Kaiser Permanente Mid-Atlantic States. We received a waiver for consent from the IRB because this was a retrospective data-only study and the data were analyzed anonymously.

## Results

### Patient characteristics

**Fig 1:**
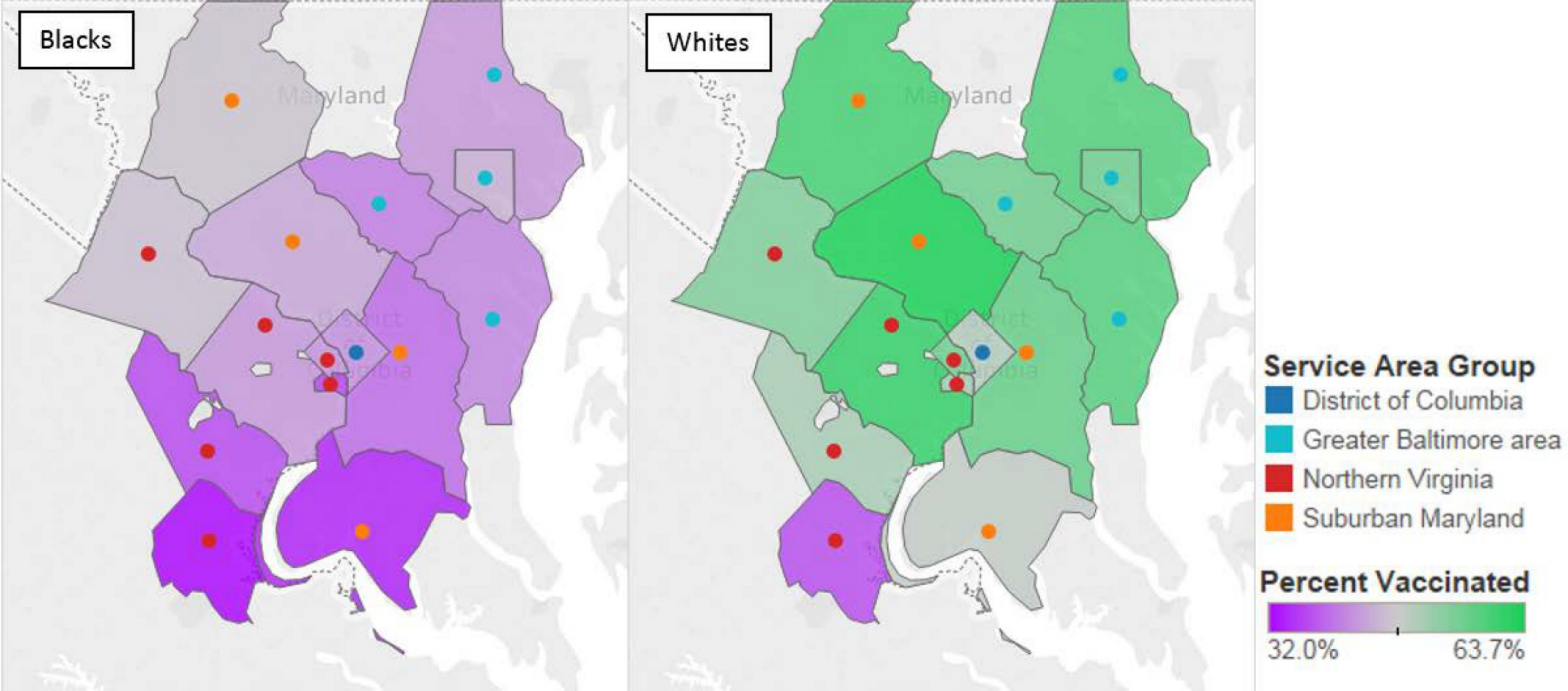
Geographic distribution of the influenza vaccination at KPMAS by race, 2014–2015.

There were 236,511 KPMAS members between May 1, 2014 and April 30, 2015 who were included in our analysis. Fig 1 shows the geographic distribution of influenza vaccinations in the four main areas served by KPMAS by race. In every service area and virtually every county, Blacks had a lower rate of vaccination than Whites. Service areas at KPMAS are administratively and geographically defined, thus, in order to provide context for results and estimates related to service area we have included a Supplemental table describing the characteristics of the 3 service areas.

Overall, our study included 129,517 (55%) Black and 106,994 (45%) White patients. The differences in characteristics/covariates between Whites and Blacks are reported in Table 1. Of Blacks, 36% were vaccinated, and of Whites, 53% were vaccinated. All of the White-Black differences were significant. These racial differences in characteristics are the key estimates to inform the **covariate effects** for the OB analysis presented later.

**Table 1:** Characteristics of KPMAS members between 5/1/14 and 4/30/15, by race.

	White (N = 106,994)	Black (N = 129,517)	White-Black Difference	P-value
Categorical Variables, N (%)				
Received influenza vaccination	56332 (52.65)	47095 (36.36)	0.1629	< 0.0001
Female	57046 (53.32)	76704 (59.22)	-0.0591	< 0.0001
Male	49948 (46.68)	52813 (40.78)	0.0591	< 0.0001
Service area			0.0544	< 0.0001
Greater Baltimore area	19324 (18.23)	16515 (12.79)	-0.0933	< 0.0001
District of Columbia	6908 (6.52)	20472 (15.85)	0.3472	< 0.0001
Northern Virginia	53875 (50.82)	20795 (16.10)	-0.3083	< 0.0001
Suburban Maryland	25905 (24.44)	71376 (55.26)	0.0121	< 0.0001
Non-current smoker	82700 (93.57)	99035 (92.35)	-0.0121	< 0.0001
Current smoker	5687 (6.43)	8199 (7.65)	-0.1806	< 0.0001
Not registered on KP.org	18934 (17.70)	46308 (35.75)	0.1806	< 0.0001
Registered on KP.org	88060 (82.30)	83209 (64.25)	0.0189	< 0.0001
CCI = 0	71271 (66.61)	80903 (62.47)	0.0415	< 0.0001
CCI = 1	17434 (16.29)	23794 (18.37)	-0.0208	< 0.0001
CCI ≥ 2	18289 (17.09)	24820 (19.16)	-0.0207	< 0.0001
Concordant patient/provider race	38853 (38.51)	46255 (37.62)	0.0089	< 0.0001
Discordant patient/provider race	62041 (61.49)	76713 (62.38)	-0.0089	< 0.0001
Continuous variables, Mean (SD)				
Age	51.39 (17.99)	48.29 (17.26)	3.1012	< 0.0001
Median household income in neighborhood (10K)	10.73 (4.06)	7.62 (3.32)	3.1053	< 0.0001
Distance to KP medical center (miles)	6.33 (5.40)	5.65 (4.30)	0.6779	< 0.0001

KPMAS = Kaiser Permanente Mid-Atlantic States; CCI = Charlson Comorbidity Index

### Odds of influenza vaccination

The race-adjusted logistic regression model estimating the odds of influenza vaccination associated with select characteristics are given in Table 2. Black members had 43% lower odds of receiving the influenza vaccination compared to White members. All of the covariates listed in Table 2, with the exception of living in DC service area, were statistically significant in terms of their association with influenza vaccination after accounting for race. Fully adjusted race-stratified logistic regression models for the odds of influenza vaccination are given in Table 3 to describe the differences in effects of characteristics between Blacks and Whites. For example, the higher odds of vaccination associated with older age and being registered on KP.org was stronger among Whites versus Blacks. The differences in the effect of characteristics on influenza vaccination between Whites and Blacks are the key estimates to inform the **coefficient effects** for the OB analysis presented later in Table 4.

**Table 2:** Race-Adjusted Odds (95% confidence interval [CI]) of Influenza Vaccination.

	Odds Ratio (95% CI)	Reference Category
Race/Ethnicity—Black	0.57 (0.56, 0.58)	White
Age (per 10 years)	1.45 (1.44, 1.46)	—
Female	1.17 (1.14, 1.19)	Male
Service area		
Greater Baltimore area	1.19 (1.15, 1.23)	Northern Virginia
District of Columbia	0.98 (0.95, 1.02)	Northern Virginia
Suburban Maryland	1.02 (1.00, 1.05)	Northern Virginia
Current smoker	0.76 (0.73, 0.79)	Non-current smoker
Registered on KP patient portal	1.57 (1.53, 1.60)	Not registered on KP.org
Neighborhood median income (per $10,000)	1.03 (1.02, 1.03)	—
Miles to nearest medical center (per mile)	0.98 (0.98, 0.98)	—
CCI = 1	1.59 (1.55, 1.63)	CCI = 0
CCI ≥ 2	2.21 (2.15, 2.28)	CCI = 0
Discordant Patient/provider race	1.07 (1.05, 1.09)	Concordant Patient/provider race

KPMAS = Kaiser Permanente Mid-Atlantic States; CCI = Charlson Comorbidity Index

**Table 3:** Odds Ratios (95% confidence interval [CI]) for Influenza Vaccination stratified by Black and White KPMAS patients.

	White	Black	
	Odds Ratio (95% CI)	Odds Ratio (95% CI)	Reference Category
Age (per 10 years)	1.49 (1.48, 1.51)	1.42 (1.41, 1.44)	—
Female	1.20 (1.17, 1.24)	1.15 (1.12, 1.18)	Male
Service Area			
Greater Baltimore area	1.16 (1.10, 1.21)	1.24 (1.17, 1.31)	Northern Virginia
District of Columbia	1.06 (1.00, 1.13)	0.89 (0.85, 0.94)	Northern Virginia
Suburban Maryland	1.18 (1.13, 1.22)	0.94 (0.90, 0.98)	Northern Virginia
Current smoker	0.67 (0.63, 0.72)	0.84 (0.80, 0.88)	Non-current smoker
Registered on KP patient portal	1.79 (1.71, 1.87)	1.46 (1.42, 1.50)	Not registered on KP.org
Neighborhood median income (per $10,000)	1.03 (1.03, 1.03)	1.02 (1.01, 1.02)	—
Miles to nearest medical center (per mile)	0.98 (0.97, 0.98)	0.99 (0.99, 0.99)	—
CCI = 1	1.49 (1.43, 1.55)	1.69 (1.63, 1.75)	CCI = 0
CCI ≥ 2	1.92 (1.83, 2.01)	2.46 (2.38, 2.56)	CCI = 0
Discordant Patient/provider race	0.93 (0.91, 0.96)	1.20 (1.17, 1.23)	Concordant Patient/provider race

KPMAS = Kaiser Permanente Mid-Atlantic States; CCI = Charlson Comorbidity Index

**Table 4:** Oaxaca-Blinder decomposition analysis for the racial disparity in influenza vaccination between Black and White* KPMAS patients.

Effect estimate**		Probability of Vaccine	SE	P-value	Lower 95% CL	Upper 95% CL	Percentage of Total Difference
**PANEL A:**	White	0.5793	0.0017	0.0000	0.5759	0.5826	
**Overall effect**	Black	0.4082	0.0015	0.0000	0.4052	0.4112	
	Total difference	0.1711	0.0023	0.0000	0.1666	0.1756	100.00
	Difference-in-covariates	0.0503	0.0022	0.0000	0.0460	0.0546	29.42
	Difference-in-coefficients	0.1208	0.0030	0.0000	0.1149	0.1266	70.58
**PANEL B:**	Age (per 10 years)	0.0280	0.0008	0.0000	0.0265	0.0294	16.34
**Covariate effect**	Female	-0.0025	0.0002	0.0000	-0.0030	-0.0021	-1.47
	Service area						
	Greater Baltimore area	0.0012	0.0002	0.0000	0.0008	0.0016	0.72
	District of Columbia	-0.0012	0.0006	0.0558	-0.0025	0.0000	-0.71
	Suburban Maryland	-0.0103	0.0012	0.0000	-0.0127	-0.0080	-6.05
	Current smoker	0.0011	0.0001	0.0000	0.0008	0.0013	0.64
	Registered on KP.org	0.0214	0.0008	0.0000	0.0198	0.0230	12.51
	Neighborhood median income (per $10,000)	0.0190	0.0013	0.0000	0.0163	0.0216	11.09
	Miles to nearest medical center	-0.0022	0.0002	0.0000	-0.0026	-0.0019	-1.31
	CCI = 1	-0.0016	0.0002	0.0000	-0.0020	-0.0013	-0.95
	CCI ≥ 2	-0.0027	0.0003	0.0000	-0.0032	-0.0021	-1.55
	Discordant patient/provider race	0.0003	0.0001	0.0001	0.0001	0.0004	0.16
**PANEL C:**	Age (per 10 years)	0.0500	0.0075	0.0000	0.0353	0.0646	29.21
**Coefficient effect**	Female	0.0060	0.0028	0.0312	0.0005	0.0115	3.51
	Service area						
	Greater Baltimore area	-0.0019	0.0010	0.0501	-0.0037	0.0000	-1.09
	District of Columbia	0.0061	0.0014	0.0000	0.0033	0.0088	3.54
	Suburban Maryland	0.0267	0.0033	0.0000	0.0203	0.0332	15.63
	Current smoker	-0.0036	0.0007	0.0000	-0.0049	-0.0023	-2.11
	Registered on KP.org	0.0290	0.0038	0.0000	0.0216	0.0364	16.95
	Neighborhood median income (per $10,000)	0.0211	0.0051	0.0000	0.0111	0.0311	12.34
	Miles to nearest medical center	-0.0149	0.0029	0.0000	-0.0206	-0.0092	-8.71
	CCI = 1	-0.0052	0.0011	0.0000	-0.0075	-0.0030	-3.06
	CCI ≥ 2	-0.0114	0.0014	0.0000	-0.0142	-0.0087	-6.67
	Discordant patient/provider race	-0.0337	0.0029	0.0000	-0.0393	-0.0280	-19.67
	Intercept	0.0526	0.0128	0.0000	0.0274	0.0777	30.72

KPMAS = Kaiser Permanente Mid-Atlantic States; SE = standard error; CCI = Charlson Comorbidity Index; CL = confidence limit

*White is the reference group

**The "Covariate effect" represents differences in probability of vaccination due to covariate values; the "Coefficient effect" represents differences in probability of vaccination due to other effects, including discrimination and unmeasured covariates.

### Oaxaca-Blinder decomposition method

Table 4 shows the results of the Oaxaca-Blinder decomposition analysis for the disparity in the probability of influenza vaccination between Blacks and Whites. In Panel A of Table 4, the overall decomposition of the disparity is shown, where the aggregated effect of the average values of the study covariates (the covariate effect) accounted for 29% of the total disparity; and the coefficient effect accounted for 71% of the total overall disparity in vaccination between Blacks and Whites. These results indicate that the racial disparity in vaccination could be reduced by 29% if the Black population becomes similar to the White population with respect to all of the average covariate values in the model. The remainder of the disparity (71%) is due to: the difference in the impact of the covariates on influenza vaccination (the coefficient effect), and/or covariates not included in the model, and/or non-linear terms not included in the model.

### Covariate effects

In Panel B of Table 4, the estimated covariate effects of each individual covariate on the probability of vaccination are reported, as well as their estimated contributions to the overall disparity in vaccination rates between Black and White members. Earlier in Table 1, we showed the White-Black difference in the proportion of members who were registered on KP.org (Black = 64% and White = 82%). With the OB analysis we are able to say that 12.5% of the total White-Black disparity in vaccinations can be attributed to the difference in registration on KP.org between Blacks and Whites (Table 4).

Conversely, the White-Black difference in the proportion of members living in Suburban Maryland (Black = 55% and White = 24% in Table 1) contributed to reducing the total White-Black disparity in vaccination by 6.0% (Table 4). Living in Suburban Maryland, though, is not a modifiable variable, therefore interpreting the meaning of the specific estimate in the context of this current OB analysis may not be practical.

Among all the covariates in Table 4, age had the largest contribution to the total White-Black disparity in vaccination, accounting for 16% of the disparity. However, age is also a non-modifiable variable. Other covariates with sizable contributions to the total White-Black disparity in vaccination were registration on kp.org, the patient website portal, (with a 13% share) and neighborhood median income (with an 11% share).

### Coefficient effects

In Panel C of Table 4, the coefficient effects on the vaccination probabilities are reported. These are the detailed decomposition of the disparity contributed by the differences in each of the estimated coefficients (i.e. odds ratios) in the logistic regression models summarized in Table 3. The estimated odds ratios of receiving vaccination associated with registering on KP.org vs. not registering on KP.org were OR = 1.79 in Whites and OR = 1.46 in Blacks (Table 3). The effect of being registered on KP.org contributed to a 17% share of the total White-Black disparity in vaccination (Table 4). In other words, the effect of being registered on KP.org on influenza vaccination is different for Blacks compared to Whites.

Among all the coefficient effects in Table 4, age, registration on KP.org, living in Suburban Maryland, and neighborhood median household income had the most sizable contributions to exacerbating the total White-Black differences/disparity in vaccination, accounting for 29%, 17%, 16%, and 12%. The constant (or intercept) contributed a 31% share of the total White-Black disparity in vaccination. The share of the constant term demonstrates that 31% of the racial disparity in vaccination was not explained by the observed covariates included in the stratified regression model.

We also conducted a sensitivity analysis where we added health care utilization variables to our models (including in-patient, out-patient, emergency department, and PCP visits). The resulting estimates with the utilization variables demonstrated that the overall covariate effects explain 35% of the total disparity rather than 29% when excluding the utilization variables. The covariate and coefficient effects of the individual variables were largely consistent. The greatest difference is that the proportion of the total disparity attributed to the coefficient effect of the intercept increased from 31% to 50%. This is largely due to the 24% decrease in the total disparity attributed to the coefficient effect of PCP visit that was included. All other differences in individual covariate or coefficient effects changed by less than 5% of the total disparity.

## Discussion

In our study of 236,511 adult, KPMAS members, 44% received the influenza vaccine between May 1, 2014 and April 30, 2015. Black members had 42% lower odds of receiving an influenza vaccination than White members. Covariates (including smoking, being registered on KP.org, and discordance of patient-provider race) had a differential effect on vaccination based on race. However, the aggregated effect of the differences in the average values of the study covariates accounted for only 29% of the total White-Black disparity. The remaining 71% of the White-Black disparity was due to the differential impact of the covariates on vaccination as well as the effect of unmeasured covariates. In other words, the disparity in influenza vaccination between Black and White KPMAS members could, at most, be reduced by 29% if all of the average covariate values in Blacks became equivalent to those of White members. We identified certain modifiable variables that could be acted upon to reduce the White-Black disparity in vaccination at KPMAS such as registration on KP.org as a possibly effective system target to reduce the racial disparity in vaccination at KPMAS. However, also understanding how covariates with differential impacts on influenza vaccination by race (the coefficient effect) contribute to racial disparities in vaccination is important to provide information to better understand the mechanisms through which the racial disparities operate (even though these effects cannot be acted on directly).

Yoo et al., were the first to apply the Oaxaca-Blinder decomposition method to assess racial/ethnic disparities in influenza vaccination rates. In their formative paper, they used the Medicare Current Beneficiary Survey (MCBS) to assess the relative contribution of a selection of covariates. They applied the Oaxaca-Blinder decomposition method to assess the relative contribution of various covariates available in the MCBS data. In our study, we followed the same methodology to assess the contribution of covariates that were available in the KPMAS electronic medical records. Our population included patients aged 18–105 (mean age = 50 years) and our data included several additional covariates that were not included in Yoo et al.’s study, including distance to medical center, Charlson comorbidity index, registration on a patient portal website, smoking, and patient-provider race discordance. Of these, only being registered on the patient portal website contributed a sizable proportion of the total racial disparity in influenza vaccinations. Yoo et al. found that 42% of the disparity in influenza vaccination between Black and White persons could be eliminated by equalizing the average values of the covariates in their regression model, while we found 29%. This difference in estimates is likely attributed to the difference in populations (eg. Medicare beneficiaries versus patients of wide ranging ages) and the type of data that was used (survey data versus real-world EMR data). Similar to Yoo et al., we found that much of the White-Black disparity in vaccination could be attributed to social demographic factors like income. We additionally found factors such as age and being registered on KP.org made significant contributions to the disparity.

As pointed out by Yoo and others [9–13], many previous studies identified racial disparities in influenza vaccinations. However, knowing which specific modifiable factors are sizable contributors to the racial disparity is especially meaningful for health care management and quality programs that aim to implement strategies to reduce disparities (especially at the health system level). Prioritizing factors that may have the biggest impact on the disparities will allow health systems to target resources efficiently and effectively. Our study indicates that one strategy to address racial disparities in vaccination at KPMAS would be to encourage Black members to utilize KP’s patient portal website. Further, our study showed that the effect of KP.org on vaccination was different for Blacks and Whites suggesting that perhaps the way the information is presented is not resonating with Blacks as it is with Whites.

The coefficient effects, because they estimate the differential impact of covariates on influenza vaccination rates by race, are considered to contribute to the unexplained gap between Blacks and Whites. However, they provide clues as to what might be contributing to the unexplained gap and allow us to make conjectures about these factors. For example, our finding of a large coefficient effect of age suggested that the impact of age on flu vaccination is different by race. It is possible that we could reduce the racial disparity in vaccinations by targeting older Black patients.

Finally, by quantifying the contributions to the racial disparity that cannot be explained by the variables in the regression model, we are able to identify gaps in our knowledge about the relationship between race and influenza vaccinations. It is clear from the results of our study that there are other factors (such as behavioral, psychosocial, lifestyle, and environmental factors) that we did not measure that could be contributing to the White-Black disparity in vaccination. Future research needs to focus on clarifying which additional contributors may be involved.

The main limitation of this study is not having behavioral or psychosocial factors that may contribute to the disparity. These may include such variables as exercise, diet, patient knowledge and attitudes towards vaccination, patient activation, etc. In addition, when we modeled our covariates, we did not include non-linear or interaction effects which may or may not have contributed to OB covariate effects. Finally, because our data represented mostly insured, employed, patients from one geographic region in the U.S., and because we restricted our analysis to only African-American and White patients, the generalizability of our results is limited to that specific population.

In conclusion, we explained the contribution of specific variables to the White-Black disparity in influenza vaccination. In terms of addressing the disparities in a health system such as KPMAS, we identified potential areas to target resources, including encouraging Black members to use the KP patient portal. However, further research should be done to identify additional factors that may contribute to racial disparities in influenza vaccinations.

## Supporting information

S1 Text Description of a hypothetical counterfactual population and an informative example. (DOC)

S1 Table Characteristics of geographic service areas of the health system population. (DOC)
