# Supplementary material for: Factors contributing to racial disparities in influenza vaccinations
Source: PLoS One. 2019 Apr 3;14(4):e0213972. doi: 10.1371/journal.pone.0213972 (PMC6447231; doi:10.1371/journal.pone.0213972)
Supplement: S1 Text — (DOC) [file pone.0213972.s001.doc]

**S1 Text. Description of a hypothetical counterfactual population and an informative example.**

To aid in the understanding of how the OB analysis decomposes the racial disparity, we constructed a hypothetical counterfactual population that can be described as having: (1) the same average covariate values as in the Black group; and (2) the same coefficient values as the White group. Using the OB methodology, the **covariate effect** is the comparison of the Whites to the hypothetical population; and the **coefficient effect** is the comparison of the Blacks to the hypothetical population. For example, suppose we wish to know how much of the difference in the proportion of patients vaccinated between Black and White patients is attributable to the difference in average income between the two groups (ie. the covariate effect of income). This quantity is estimated by first comparing the White group to the hypothetical population having the same coefficient values, but the Black average covariate values, thus giving the overall covariate effect. We then estimate the contribution of income to the overall covariate effect. Similarly, suppose we wish to know how much of the racial difference in patients vaccinated is attributable to the difference in the impact of income on vaccination rates between the two groups (ie. the coefficient effect of income). This quantity is estimated by first comparing the Black group to our hypothetical population having the same average covariate values, but the White coefficient values, thus giving the overall coefficient effect. We then compute the contribution of income to the overall coefficient effect. The estimates of interest for the OB models are the White-Black difference in the probability of vaccination, and the percentages of the contributions of the covariates (as well as the effects of those covariates) on the disparity in vaccinations.
