# Supplementary material for: Factors contributing to racial disparities in influenza vaccinations
Source: PLoS One. 2019 Apr 3;14(4):e0213972. doi: 10.1371/journal.pone.0213972 (PMC6447231; doi:10.1371/journal.pone.0213972)
Supplement: S1 Table — (DOC) [file pone.0213972.s002.doc]

**S1 Table. Characteristics of Geographic Service Areas of the Health System Population**

|  | **Balt** | **DCSM** | **NOVA** |
| --- | --- | --- | --- |
| Number of patients | 127,612 | 335,165 | 294,363 |
| Female, % | 52.5 | 53.8 | 52.0 |
| Insurance type, % |  |  |  |
| Commercial | 67.6 | 75.8 | 85.6 |
| Medicare | 11.8 | 10.8 | 7.8 |
| Medicaid | 19.6 | 11.9 | 5.2 |
| Other | 0.8 | 0.1 | 1.2 |
| Race/ethnicity, % |  |  |  |
| White | 41.1 | 20.3 | 39.0 |
| Black | 40.5 | 53.0 | 17.3 |
| Hispanic | 5.7 | 14.5 | 18.1 |
| Asian/PI | 10.7 | 9.7 | 22.0 |
| AI/AN | 0.19 | 0.17 | 0.16 |
| Multi | 1.8 | 2.4 | 3.3 |
| Median age | 39 | 40 | 38 |
| Median household income* | 69,556 | 83,582 | 102,439 |
| Median miles to MC | 5.3 | 4.3 | 5.0 |
| Medical center density  (per 100,000 patients) | 7.8 | 6.0 | 4.8 |
| Patient to Physician density  (per 100,000 patients) | 189.6 | 207.7 | 181.4 |

Balt = Greater Baltimore region; DCSM = District of Columbia and suburban Maryland; NOVA = Northern Virginia; PI = Pacific Islander; AI = American Indian; AN = Alaskan Native

*Neighborhood tract-level variable
